# The peer review process: Growing problem in recruiting qualified reviewers

**DOI:** 10.2340/aos.v83.42370

**Published:** 2024-11-21

**Authors:** Palle Holmstrup

**Affiliations:** Faculty of Health and Medical Sciences, Department of Odontology, Section for Oral Biology and Immunopathology, Copenhagen N, Denmark

Everyone publishing in health science journals knows that the peer review process is extremely important to ensure the scientific quality of published articles. Nevertheless, in recent years it has become increasingly difficult to recruit qualified reviewers. The consequence is that the editorial process becomes more time-consuming, and frustrated authors are asking about the progress of their manuscripts.

In the editorial office of Acta Odontologica Scandinavica we have experienced the growth in declining invited potential reviewers. While the average number of invited reviewers was 2–3 per submission in the period 2006-2010, recent data show that during 2019–2024 the average number of invited reviewers per submitted manuscript is 6–7. Moreover, nowadays it is not uncommon to invite 20 researchers or more to recruit 2 accepting reviewers.

The situation, which appears to be a common problem for scientific journals in the health science sector, has recently been addressed by the Editor-in-Chief of Journal of the European Academy of Dermatology and Venereology, Professor Carle Paul^1^. In an important Editorial he demonstrates the size of the problem by showing the increasing number of declining invited reviewers in the period 2020 to 2023. As an appeal to the community of researchers he argues that based on the law of reciprocity, there is an obligation to repay for *“all of us who have had the opportunity to experience a fruitful academic career. Just as we are grateful to our mentors who guided us through the complexities of scientific research, we are indebted to the hundreds of reviewers who volunteered their time to review our manuscripts.”* Also, the author mentions that *“junior researchers are often genuinely motivated to participate in peer review and have more time to devote to this activity. In addition, they tend to be very knowledgeable in their field”,* which may also increase the sustainability of the process and improve young researchers’ writing skills and stimulate formation of ideas for future research^1^.

These clever words are important to notice, and they call for the essential understanding of the reciprocity in giving and taking benefit – two processes that are inseparable in a fair and developing scientific community.

There appears to be no obvious ways to force researchers to act as peer reviewers. The only way to improve the current situation is to appeal to the researcher’s conscience, which is hereby done.

## Footnotes

1 Paul C. Peer reveview, scientific publishing and the law of reciprocity. J Eur Acad Dermatol Venereol 2024; 38: 988–9.

